# Attention-deficit/hyperactivity disorder symptoms and subsequent cardiometabolic disorders in adults: investigating underlying mechanisms using a longitudinal twin study

**DOI:** 10.1186/s12916-023-03174-1

**Published:** 2023-11-22

**Authors:** Maja Dobrosavljevic, Ralf Kuja-Halkola, Lin Li, Zheng Chang, Henrik Larsson, Ebba Du Rietz

**Affiliations:** 1 School of Medical Sciences, Faculty of Medicine and Health, Örebro University, SE-701 82 Örebro, Sweden; 2 Department of Medical Epidemiology and Biostatistics, Karolinska Institutet, Stockholm, Sweden

**Keywords:** ADHD, ADHD symptoms, Cardiometabolic disorders, Twin study, Longitudinal study, Adults, Underlying mechanisms, Genetics, Comorbidity

## Abstract

**Background:**

Emerging research suggests that attention-deficit/hyperactivity disorder (ADHD) increases the risk for cardiovascular (CVDs) and metabolic disorders (i.e., cardiometabolic disorders) in adulthood. Yet, available studies are scarce and have mainly been focused on individuals receiving clinical ADHD diagnoses. We aimed to investigate the prospective associations of ADHD symptoms in young and mid-adulthood with subsequent cardiometabolic disorders and the underlying mechanisms.

**Methods:**

We studied 10,394 twins from the Swedish Twin Registry (STR), born between 1958 and 1985 without previous medical history of cardiometabolic disorders. They provided self-assessment of ADHD symptoms (score range 0–36) via a validated, DSM-IV-based scale in a web-based questionnaire/telephone interview within the Study of Twin Adults: Genes and Environment (STAGE), in 2005–2006 (aged 19–47 years), and were followed until the end of 2018 (33–59 years) to identify incident clinical diagnoses/medication prescriptions for cardiometabolic disorders acquired from Swedish national registers. We used Cox regression models to investigate the associations between ADHD symptoms score and cardiometabolic outcomes, with and without adjustment for relevant covariates, and a co-twin control design to study familial confounding.

**Results:**

A one-unit increase in the level of ADHD symptoms was associated with a 2% increase in the rate of CVDs (hazard ratio [HR] = 1.02, 95% confidence interval 1.01–1.04) and a 3% increase in the rate of metabolic disorders (HR = 1.03, 1.02–1.05), after adjusting for birth year and sex. The associations were no longer significant after adjusting for educational attainment, lifestyle factors, and comorbid psychiatric disorders. The associations remained significant after adjusting for familial factors shared by dizygotic twin pairs but became nonsignificant after adjusting for factors shared by monozygotic twin pairs. However, the strength of the associations attenuated significantly in monozygotic twins compared to dizygotic twins for CVDs only, suggesting genetic confounding.

**Conclusions:**

ADHD symptom score is associated with a higher risk for cardiometabolic disorders, which may be explained by lower educational attainment, adverse lifestyle factors, and psychiatric comorbidities. Moreover, the associations appear to be partly confounded by shared genetic factors, especially for CVDs. Further research is needed to investigate the identified associations at the level of individual cardiometabolic disorders and to follow-up participants until a more advanced older age.

**Supplementary Information:**

The online version contains supplementary material available at 10.1186/s12916-023-03174-1.

## Background

Attention-deficit/hyperactivity disorder (ADHD) is a neurodevelopmental condition that typically emerges in childhood and is characterized by inattentive and/or hyperactive symptoms that often persist well into adulthood [1, 2]. While comorbid mental health problems have been well documented in adults with ADHD, less is known on physical health problems, especially in relation to age-related disorders, such as cardiovascular disorders (CVDs) (i.e., ischemic heart disease, heart failure, cerebrovascular disease, venous thromboembolism, tachyarrhythmias, and hypertension), and common metabolic disorders (i.e., type 2 diabetes, obesity, and hyperlipidemia) [3]. CVDs are the leading cause of death and one of the major causes of disability in adults globally [4], and the rising burden of metabolic disorders has become a major healthcare concern worldwide [5, 6].

Emerging research has reported significant associations of ADHD with increased risk for a range of CVDs and metabolic disorders (i.e., cardiometabolic disorders), both in children [7] and adults [8–16]. Yet, available large-scale studies that have reported an increased risk of cardiometabolic disorders in adults have been limited to definitions of ADHD based on clinical diagnoses using health record data [9, 10, 12, 14]. This may be problematic in adults, and especially in older adults, in whom ADHD is likely underdiagnosed [1]. Older individuals may experience adverse health outcomes due to elevated ADHD symptoms without receiving adequate health care [17], and the population diagnosed with ADHD may not cover all individuals affected by the symptoms. Additionally, there have only been a few longitudinal studies investigating the associations between ADHD and cardiometabolic disorders, and these studies have had follow-up periods limited to early adulthood [11, 12, 18]. Longitudinal studies that follow individuals until a more advanced age are needed to establish the long-term associations of ADHD with subsequent cardiometabolic disease.

There is also a scarcity of research investigating the underlying mechanisms of the potential associations between ADHD and cardiometabolic disorders. Two potential mechanisms have been proposed. First, ADHD is associated with several adverse life-course outcomes, such as lower socioeconomic status, lifestyle factors, and comorbid psychiatric disorders, which are also risk factors for cardiometabolic disorders [19, 20]. Indeed, recent studies have pointed towards life-course risk factors (i.e., socio-economic variables, lifestyle factors, and psychiatric comorbidities) that may partially mediate the association between ADHD genetic liability (i.e., a proxy measure of ADHD risk) [13] or ADHD diagnoses [10, 14, 21] and the risk of cardiometabolic disorders. However, none of these studies have considered the associations of ADHD symptoms severity with cardiometabolic disorders. Second, a limited number of genetic studies, using family-based and molecular genetic designs, have recently suggested weak-to-moderate genetic correlations between ADHD and several cardiometabolic outcomes [9, 22, 23]. However, it remains unknown whether the associations between ADHD and cardiometabolic outcomes are confounded by familial or genetic factors. Considering the weak sharing of genetic factors, it seems plausible that the increased risk of cardiometabolic disorders in ADHD may be largely explained by environmental risk factors or bidirectional causal effects. A 2-sample Mendelian randomization study indicated that ADHD may have a causal effect on childhood obesity and coronary artery disease but found limited evidence for inferring causal effects on other cardiometabolic disorders [24]. Further research is needed to determine the increased risk of cardiometabolic diseases in ADHD and to establish to what extent educational attainment, lifestyle, and psychiatric comorbidities, as well as shared underlying genetic factors, explain the associations. It is crucial to improve our understanding of the increased risk of cardiometabolic diseases in ADHD and to identify potentially modifiable risk factors, in order to guide clinical practice and improve prevention strategies that promote the health of aging individuals with ADHD.

In this study, we employed a longitudinal population-based study to investigate the associations of ADHD symptoms severity in young- and mid-adulthood with subsequent cardiometabolic disorders in later life. We further investigated whether any identified associations were explained by educational attainment, lifestyle factors, and comorbid psychiatric disorders or confounded by familial factors (genetic and environmental factors shared by twins), using a genetically informed co-twin control approach.

## Methods

### Study population and main measurements

A total of 42,582 twins born in Sweden between 1959 and 1985 who survived their first birthday were identified from the population-based Swedish Twin Registry [25]. Out of this population, 25,321 (59.5%) individuals participated in the Study of Twin Adults: Genes and Environment (STAGE) [26]. From 2005 to 2006, participants responded to questions in relation to their physical and mental health, lifestyle, and socioeconomic/demographic factors via a web-based questionnaire or telephone interview, which contained 1300 questions. Non-responders did not differ from responders with regards to age, but they were significantly more likely to be male, to have at least one parent born outside Sweden, and to be diagnosed with a psychiatric disorder [26, 27].

**Fig. 1:**
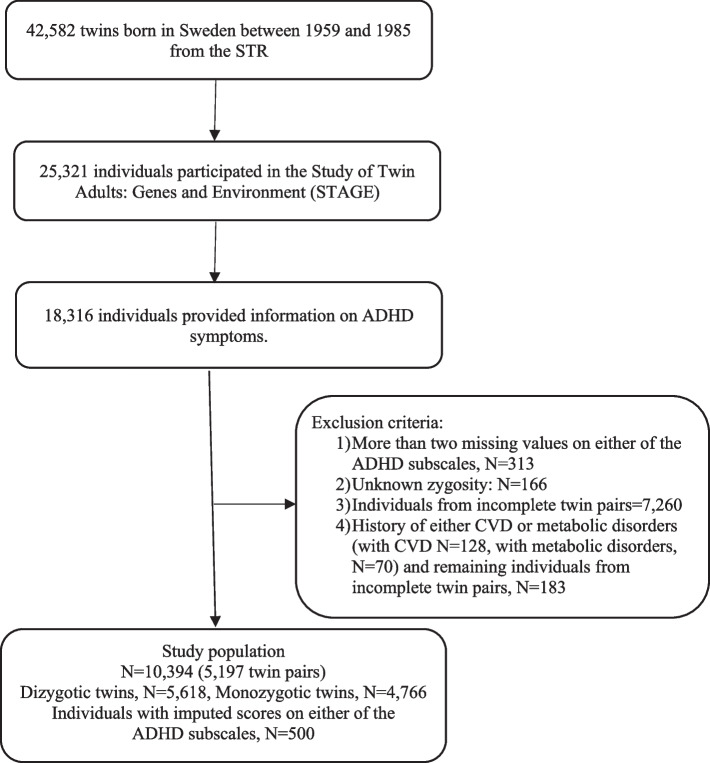
Flowchart of the study population selection process

In total, 18,316 individuals provided information on ADHD symptoms, among which 40% (*N* = 7366) were men [26]. Self-reported ADHD symptoms were assessed via nine inattention items and nine hyperactivity/impulsivity items in accordance with DSM-IV diagnostic criteria for ADHD and slightly modified to fit adults [26]. Each item had a three-point answer format, with 0 = ”No,” 1 = ”Yes, to some extent,” and 2 = ”Yes.” The symptoms were summed into two subscales of ADHD symptoms: inattention and hyperactivity/impulsivity. A validation study of the scale was conducted earlier [26], with both subscales showing good internal consistency with Cronbach *α* of 0.79 for inattention and 0.77 for hyperactivity/impulsivity. In case there were more than two missing items for either of the two subscales, a participant would be deleted from the analysis, and when there were two or fewer missing values, missing items were replaced with a mean score for this subscale (see Fig. 1 for the number of excluded individuals and the number of individuals with imputed scores) [28]. We considered the total score for the analysis (i.e., the sum of scores on the inattention and hyperactivity/impulsivity subscales, total score range 0–36).

Information on zygosity was obtained via standard physical similarity questions which have been previously validated through genotyping [29]. We excluded individuals from incomplete twin pairs (see Fig. 1 for the number of excluded individuals).

We included the following incident CVDs: ischemic heart disease, heart failure, cerebrovascular disease, venous thromboembolism, tachyarrhythmias, and hypertension; and metabolic disorders: type 2 diabetes, obesity, and hyperlipidemia. All CVDs and metabolic disorders were defined as diagnoses obtained from the National Patient Register, coded following the International Classification of Diseases 10th revision (ICD-10) or dispensed medication prescriptions from the Prescribed Drug Register (PDR) and coded according to the Anatomical Therapeutic Classification (ATC) system (Additional file 1: Table S1). The NPR contains information on specialist care diagnoses—inpatient care diagnoses since 1987 and outpatient care since 2001 [30], while the PDR covers all dispensed medications since July 2005, including both specialist and primary health care [31]. We used information on dispensed medications for the treatment of cardiometabolic disorders from the PDR in addition to ICD-based diagnoses from the NPR, as individuals with these disorders are often diagnosed and treated within primary care and may not be covered in the NPR. The incident diagnoses/medication prescriptions were registered after the STAGE assessment and before December 31, 2018. We excluded individuals with a medical history of either CVD or metabolic disorder (either a diagnosis or dispensed medication prescription, Additional file 1: Table S1) before the STAGE interview/questionnaire (Fig. 1).

### Covariates

The following variables, previously reported to be associated with ADHD [19] and cardiometabolic disorders [20], and assessed in the STAGE interview, were considered as covariates: (i) educational attainment (compulsory/secondary level//higher education), (ii) lifestyle factors: smoking, body mass index (BMI), and physical activity, and (iii) and self-reported symptoms of psychiatric disorders: major depression, generalized anxiety disorder, and alcohol dependence. Participants were categorized into three categories in relation to smoking: never/just tried smoking, smoking occasionally, and regularly smoking [32]. BMI was calculated (kg/m^2^) based on self-reported weight and height and classified in accordance with the WHO criteria into four categories: underweight (< 18.5 kg/m^2^), normal weight (18.5–24.9 kg/m^2^), overweight (25.0–29.9 kg/m^2^), and obese (≥30 kg/m^2^) [33]. Participants rated their physical activity on a scale from 1 to 10, with 1 = very low (mainly sedentary), 5 = moderate physical activity (a few walks per week), and 10 = very high (sports/jogging several times a week). The scale was collapsed into the following four categories: 1–4 (sedentary/low), 5–6 (moderate), 7–8 (high), and 9–10 (vigorous) [34].

Criteria based on the Diagnostic and Statistical Manual of Mental Disorders, fourth revision (DSM-IV), were used for self-reported symptoms of major depression, generalized anxiety disorder, and alcohol dependence [27]. Detailed descriptions of the criteria are provided in Additional file 1: Supplementary methods.

### Data analysis

#### Phenotypic analyses

We used Cox proportional hazards models to assess the associations between ADHD symptoms and subsequent CVDs and metabolic disorders during the follow-up, with attained age as the underlying time scale. Associations were presented as hazard ratios (HRs) with 95% confidence intervals (CIs). The follow-up period started at the time of the interview/questionnaire completion and ended at either a diagnosis/dispensed medication prescription of the outcome of interest, death, or December 31, 2018. A cluster robust sandwich estimator was used to remove distributional assumptions and correct CIs for dependence on family data. The analysis was adjusted for sex and birth year in the first step, and then additionally adjusted for educational attainment, lifestyle factors, and comorbid psychiatric disorders in the second step.

#### Genetically informed analyses

The co-twin control design was used to investigate whether ADHD is directly associated with CVDs and metabolic disorders after accounting for familial (genetic and/or shared environmental) factors. To control for unmeasured familial confounding, we applied a stratified Cox regression model with each twin pair being entered as a separate stratum. By using this model, we analyzed the correlation between the difference in ADHD symptoms within a twin pair and the difference in subsequent cardiometabolic outcomes within the same pair [35]. We modeled separate estimates for monozygotic twins who share 100% of their segregating genes and 100% of shared environment, with no additional adjustments for sex and age, and for dizygotic twin groups who share around 50% of segregating genes and 100% of shared environment, with adjustments for sex since we included opposite sex twins. Associations observed within monozygotic twins can be only driven by non-shared environment since this is the only factor making them dissimilar from each other, while within dizygotic twins, the association can be driven by both non-shared environment and genetics. Thus, the presence of genetic effects can be assessed by comparing the estimates from monozygotic and dizygotic twin-pair analyses using a Wald-type *χ*^2^ test statistic [35]. Due to the low incidence of individuals diagnosed with distinct categories of cardiometabolic disorders (Additional file 1: Table S2) and consequent low statistical power for the analysis, we presented the main results at the broader level of CVDs and metabolic groups of disorders.

#### Sensitivity analyses

We conducted five sensitivity analyses. First, we investigated the patterns of associations with cardiometabolic outcomes when we consider the scores on the two ADHD subscales, separately: the inattention and hyperactivity/impulsivity subscale. Second, we explored whether the associations remain the same depending on the definition of ADHD, using clinical and research-based diagnoses. Clinical diagnoses of ADHD were based on ICD codes obtained from the NPR (ICD-9 code: 314, ICD-10 code: F90), established at any time point, since we assumed that individuals with ADHD diagnoses had childhood-onset of symptoms, in line with the DSM-IV diagnostic criteria. Research diagnoses of ADHD were obtained using the norm-based criteria [36]: the primarily inattentive subtype was identified in individuals with scores 2 standard deviations (SDs) above the mean (i.e., above the 98th percentile) on the inattention subscale, but not hyperactivity/impulsivity subscale; the primarily hyperactive/impulsive subtype was identified in those with scores 2 SDs above the mean on the hyperactivity/impulsivity subscale, but not for inattention, and participants with scores 2 SDs above the mean on both subscales were considered as having the combined subtype. Third, we examined the effects of sex on the association of ADHD with cardiometabolic disorders by stratifying the analysis for men and women. Fourth, we investigated whether the adjustments for familial confounding within dizygotic twins stayed consistent when we limited our analyses to same-sex twin pairs, to match the monozygotic twin pairs who are of the same sex. Finally, as the Cox regression models assume a linear association between ADHD symptoms and cardiometabolic disorders, on the log-hazard ratio scale, we also modeled potential non-linear associations by using the natural cubic splines function for descriptive purposes [37].

Data management and analysis was done using SAS V.9.4 (SAS Institute) and R version 4.1.0.

## Results

### Descriptive characteristics of the study population

The study population was composed of 10,394 individuals, among which 3985 were male (38.3%). The mean age at the time of STAGE interviews was 33.30 years (SD = 7.68, range = 19–47), and at the end of the follow-up, it was 46.28 years (SD = 7.68, range = 33–59). There were 4766 monozygotic and 5618 dizygotic twins with 2962 individuals from same-sex twin pairs. During the follow-up period, 1313 (12.6%) individuals had an incident diagnosis or dispensed medication prescription for any CVD and 728 (7%) for any metabolic disorder (see Additional file 1: Table S2 for frequencies of individual disease categories). Scores on the ADHD scale were significantly positively skewed (*p* < 0.010) and significantly correlated with age (Pearson correlation − 0.072, *p* < 0.0001), sex, cardiometabolic outcomes, and all considered covariates (Table 1).

**Table 1:** Baseline descriptive characteristics of the study population

		*N* (%)	ADHD score	
			Mean (SD)	Difference
Total		10,394	4.60 (4.68)	*t* test = 2.00,* p* = 0.046
Male		3985 (38.34)	4.72 (4.75)	
Female		6409 (61.66)	4.53 (4.65)	
With incident CVD		1313 (12.63)	4.94 (5.26)	*t* test = − 2.78, *p* = 0.005
Without incident CVD		9081 (87.37)	4.55 (4.59)	
With incident metabolic disorder		728 (7.00)	5.25 (5.21)	*t* test = − 3.86, *p* < 0.001
Without incident metabolic disorder		9666 (93.00)	4.55 (4.64)	
Educational attainment *N* = 9507, male = 3706, female = 5801	Compulsory	434 (4.57)	6.10 (5.86)	*F* value = 56.09, *p* < 0.001
	Secondary	4062 (42.73)	4.88 (4.83)	
	Higher	5011 (52.71)	4.13 (4.28)	
Smoking *N* = 9609, male = 3491, female = 6118	Never/just tried	5758 (59.92)	4.14 (4.33)	*F* value = 74.31, *p* < 0.001
	Occasionally	1763 (18.35)	4.89 (4.80)	
	Regularly	2088 (21.73)	5.54 (5.30)	
Body mass index *N* = 10,227, male = 3962, female = 6265	Underweight	283 (2.77)	4.25 (4.34)	*F* value = 9.87, *p* < 0.001
	Normal weight	7198 (70.38)	4.47 (4.58)	
	Overweight	2230 (21.81)	4.85 (4.88)	
	Obese	516 (5.05)	5.44 (5.23)	
Physical activity *N* = 10,343, male = 3968, female = 6375	Sedentary/low	188 (18.17)	5.29 (5.15)	*F* value = 14.26, *p* < 0.001
	Moderate	2682 (25.93)	4.52 (4.59)	
	High	3523 (34.06)	4.37 (4.62)	
	Vigorous	2252 (21.77)	4.44 (4.40)	
Major depression *N* = 10,322, male = 3962 female = 6360	With	1071 (10.38)	6.77 (5.84)	*t* test = − 16.29, *p* < 0.001
	Without	9251 (89.62)	4.34 (4.45)	
Generalized anxiety disorder *N* = 10,223, male = 3931, female = 6292	With	537 (5.25)	8.30 (6.44)	*t* test = − 19.41, *p* < 0.001
	Without	9686 (94.75)	4.36 (4.45)	
Alcohol dependence, *N* = 10,157, male = 3911, female = 6246	With	808 (7.96)	7.24 (5.83)	*t* test = − 16.82, *p* < 0.001
	Without	9349 (92.04)	4.38 (4.51)	

### Associations of ADHD symptoms with CVDs and metabolic disorders

An increase of one unit in the level of ADHD symptoms (score range 0–36) was associated with a 2% increase in the rate of CVDs (HR = 1.02, 95% CI 1.01–1.04, *p* < 0.001) and a 3% increase in the rate of metabolic disorders (HR = 1.03, 95% CI 1.02–1.05, *p* < 0.001), after adjusting for birth year and sex (Table 2). The associations attenuated and became nonsignificant after adjusting for educational attainment, lifestyle factors, and comorbid psychiatric disorders (HR = 1.00, 95% CI 0.99–1.02, *p* = 0.611, for CVDs, HR = 1.01, 95% CI 1.00–1.03 1.03, *p* = 0.076, for metabolic disorders).

**Table 2:** Associations of ADHD symptom scores with cardiovascular (CVD) and metabolic disorders, presented as hazard ratios (HR) with 95% confidence intervals (CI)

	CVD HR (95% CI)		Metabolic disorders HR (95% CI)	
Adjustment for birth year and sex^a^	**1.02 (1.01, 1.04), *p*** < **0.001**		**1.03 (1.02, 1.05), *p*** < **0.001**	
Additional adjustment for covariates^b^	1.00 (0.99, 1.02), *p* = 0.611		1.01 (1.00, 1.03), *p* = 0.076	
Additional adjustment for familial factors shared between twins in:				
Dizygotic twin pairs^c^	**1.04 (1.02, 1.07), *p*** < **0.001**	***χ***^**2**^ = **3.98, *p*** = **0.045**	**1.04 (1.01, 1.07), *p*** = **0.021**	*χ*^2^ = 0.40, *p* = 0.839
Monozygotic twin pairs^d^	1.00 (0.97, 1.03), *p* = 0.968		1.04 (1.00, 1.09), *p* = 0.069	

^a^*N* = 10,394 total included in the analysis, with CVD *N* = 1313, with metabolic disorders *N* = 728

^b^Covariates: educational attainment, lifestyle, and psychiatric disorders, *N* = 8305 total included in the analysis for the association with CVD, *N* with CVD is 1007; *N* = 8434 total included in the analysis for the association with metabolic disorders, *N* with is 587, analysis for the association with metabolic disorders was not adjusted for BMI

^c^*N* = 5618 total included in the analysis, with CVD *N* = 747, with metabolic disorder *N* = 435

^d^*N* = 4766 total included in the analysis, with CVD *N* = 566, with metabolic disorders *N* = 293

Bolded estimates are statistically significant, *p*-value less than 0.001 or 0.05

### The role of familial factors for associations of ADHD symptoms with CVDs and metabolic disorders

Within the dizygotic twins, where we adjusted the analysis for familial factors shared by dizygotic twin pairs, the associations of ADHD symptoms with both CVDs (HR = 1.04, 95% CI 1.02–1.07, *p* < 0.001) and metabolic disorders (HR = 1.04, 95% CI 1.01–1.07, *p* = 0.021) remained statistically significant (Table 2). Within the monozygotic twins, where we adjusted for familial factors shared by monozygotic twin pairs, the estimates attenuated, and the associations of ADHD symptoms with CVDs (HR = 1.00, 95% CI = 0.97–1.03, *p* = 0.968) and metabolic disorders (HR = 1.04, 95% CI = 1.00–1.09, *p* = 0.069) were no longer statistically significant. The difference in estimates between monozygotic and dizygotic twins was statistically significant for CVDs (*p* = 0.045), but not for metabolic disorders (*p* = 0.839) (Table 2).

### Sensitivity analysis

When examining the ADHD subscales separately, we found significant associations of both inattention and hyperactivity/impulsivity subscale with CVDs and metabolic disorders, which attenuated and were no longer significant after adjustments for covariates (i.e., educational attainment, lifestyle factors, and comorbid psychiatric disorders). The associations remained significant after adjustments for familial factors shared between dizygotic twins across both inattention and hyperactivity/impulsivity subscales, except for the association of the inattention subscale and metabolic disorders, with HR = 1.05, 95% CI = 1.00–1.11, *p* = 0.061 (Additional file 1: Table S3). In the monozygotic twin cohort, the only association that remained statistically significant was between hyperactive/impulsive symptoms and metabolic disorders (HR = 1.09, 95% CI = 1.01–1.18, *p* = 0.027); this association was similar in magnitude to the association observed for dizygotic twins (HR = 1.06, 95% CI = 1.01–1.12, *p* = 0.031).

We also considered different definitions of ADHD, using a clinical and research diagnosis of ADHD. There were 79 (0.76%) individuals with a clinical diagnosis of ADHD and 311 (2.99%) with a research diagnosis of any ADHD subtype (Additional file 1: Table S4). The associations of ADHD based on both definitions with cardiometabolic disorders were consistent with the main findings (Table 3). Both individuals with a clinical and research diagnosis were at an increased risk of CVD and metabolic disorders, and the risk attenuated and became non-significant after adjusting for covariates. However, we could not run the co-twin control analyses due to the low statistical power.

**Table 3:** Associations of clinical and research diagnosis of ADHD with cardiovascular disorders (CVDs) and metabolic disorders, as hazard ratios with 95% confidence intervals

	CVD	Metabolic disorders
**Clinical diagnosis**		
Adjustment for birth year and sex^a^	**2.34 (1.47, 3.74), *p*** < **0.001**	**2.19 (1.20, 4.00), *p*** = **0.011**
Additional adjustment for covariates^b^	1.64 (0.91, 2.94), *p* = 0.097	1.75 (0.88, 3.45), *p* = 0.107
**Research diagnosis**		
Adjustment for birth year and sex^a^	**1.61 (1.23, 2.11), *p*** < **0.001**	**1.77 (1.27, 2.46), *p*** < **0.001**
Additional adjustment for covariates^b^	1.09 (0.77, 1.54), *p* = 0.629	1.00 (0.64, 1.55), *p* = 0.999

^a^*N* = 10,394, with CVD *N* = 1313, with metabolic disorders *N* = 728

^b^Covariates: educational attainment, lifestyle, and psychiatric disorders, *N* = 8305 for the association with CVD, *N* with CVD is 1007; *N* = 8434 for the association with metabolic disorders, *N* with metabolic disorders is 587, analysis for the association with metabolic disorders was not adjusted for BMI

Bolded estimates are statistically significant, *p*-values less than 0.001 or 0.05

Analyses stratified by sex showed that the associations of ADHD symptoms with CVDs and metabolic disorders were statistically significant across both men and women (Additional file 1: Table S5 and S6, respectively), but they remained significant after adjusting for covariates for the associations with metabolic disorders in women only. The associations with CVDs remained significant after adjustments for familial factors shared between dizygotic twins but became non-significant after adjustments for familial factors shared between monozygotic twins, across both men and women. The associations with metabolic disorders were no longer significant after adjustments for familial factors shared between dizygotic and monozygotic twins, across both sexes. Furthermore, when we limited the analysis within dizygotic twins to same-sex pairs only, only the associations with CVDs were significant (Additional file 1: Table S7). Finally, the cubic spline functions suggested linear (rather than non-linear) associations between ADHD and cardiometabolic disorders on the log-hazard ratio scale (Additional file 1: Fig. S1 and S4). The statistical power of the analysis within twin groups was likely insufficient since there were too few participants with high ADHD scores (Additional file 1: Fig. 2–3 and 5–6).

## Discussion

This is the first large population-based study to investigate associations between ADHD and cardiometabolic disorders using ADHD symptom level data and a longitudinal approach with follow-up of up to 14 years. Additionally, this is the first study to control for unmeasured familial confounding using a robust, co-twin control study design. We found that ADHD symptom severity was associated with increased risk for both CVDs and metabolic disorders; however, these associations were no longer significant after adjusting for educational attainment, lifestyle factors, and comorbid psychiatric disorders. Our results further suggested that the associations between ADHD symptoms and CVDs might be confounded by genetic factors; however, there was not as strong evidence for genetic confounding for metabolic disorders.

Our results confirmed and extended the findings of recent register/health record studies of the increased risks for CVDs and metabolic disorders in adults with clinically diagnosed ADHD [8–10, 13, 14] by showing that the risk remained elevated even at the symptom level of ADHD, in a non-clinical sample. This finding is of importance considering that register/health record data may have biased prevalence estimates since ADHD likely remains underdiagnosed in adults aged 50 years and older [1]. Some earlier studies did not identify significant associations between ADHD and CVDs and metabolic disorders [38–40]. However, these studies either had small samples, they utilized self-reported medical history of cardiometabolic disorders [38, 40], or they used medical claims from commercial databases that may not generalize to the general population [39].

We further found that the strength of the associations attenuated and did not remain significant after adjusting for educational attainment, lifestyle factors (i.e., smoking, body mass index, and physical activity), and comorbid psychiatric disorders (i.e., major depression, generalized anxiety disorder, and alcohol dependence). This indicates that the risk of cardiometabolic disorders associated with ADHD symptoms is at least partly explained by adverse socioeconomic factors, lifestyle, and psychiatric health outcomes [10, 13, 14]. These variables can be seen as potentially modifiable risk factors of cardiometabolic disorders [41]—they may be, at least partially, managed through treatment and/or behavioral changes (e.g., psycho/pharmacotherapy for psychiatric comorbidities, smoking cessation, developing healthier eating habits, and increasing physical activity levels), as opposed to non-modifiable risk factors such as age, sex, family history, and ethnicity. Thus, they could be targeted in future preventative strategies to improve long-term health outcomes in individuals with ADHD.

Furthermore, we cannot rule out the additional effects of other potentially relevant early-life factors (e.g., birth weight or preterm birth) or lifestyle factors such as sleep disorders and nutrition [4, 19, 20]. We did not include these factors in the current study as we did not have access to the relevant data for preterm birth/low birth weight and due to large amounts of missing values on the corresponding scales for sleep disorders and nutrition and the substantial reduction of the statistical power of the analyses which would occur with their inclusion. Future studies using good-quality data on these variables are needed to further investigate the effects of other potentially relevant confounders and mediators of the associations of ADHD symptoms with cardiometabolic disorders.

Additionally, the associations between ADHD symptoms and CVDs seemed to be confounded by familial factors. Our findings also suggested that these familial factors might be, at least to some extent, driven by shared genetic factors that influence both ADHD symptoms and CVDs, as evidenced by the significantly stronger attenuation of the association when controlling for familial factors shared by monozygotic twins compared to dizygotic twins. This is largely in line with a previous register-based sibling study that reported that shared familial factors partly explained the association between ADHD and CVDs [9]. Yet, the previous study found no evidence of shared genetic factors, possibly due to low statistical power due to few older adults with both clinically diagnosed ADHD and CVDs [9]. Nevertheless, a recent genome-wide study reported significant genetic correlations between ADHD and coronary heart disease [22].

For metabolic disorders, on the other hand, our results suggested potential familial confounding of the associations with ADHD, but not strong evidence for genetic confounding, in contrast to previous research [9, 23]. One reason for this inconsistency may be the different definitions of ADHD and metabolic disorders. This study is the first to investigate familial confounding underlying the association between ADHD and metabolic disorders using symptoms of ADHD as opposed to clinical diagnoses. Furthermore, we used a broad definition of metabolic conditions, including type 2 diabetes, obesity, and hyperlipidemia. A previous register-based sibling study found that results varied for different individual metabolic disorders; there was strong evidence for shared genetic factors between ADHD and obesity, but not ADHD and type 2 diabetes [9], while genome-wide association studies [22, 23] have reported small but significant genetic correlations between ADHD and obesity and type 2 diabetes. While it would have been interesting to investigate genetic confounding of the associations between ADHD and the different metabolic disorders, we were not well-powered to study disorders separately within the co-twin framework.

When examining the two ADHD subscales separately, we found that hyperactivity/impulsivity symptoms were significantly associated with metabolic disorders within monozygotic twin pairs, which indicates direct effects via non-shared environmental influences. These findings suggest that symptoms of hyperactivity/impulsivity may lead to cumulative, health-adverse effects throughout adulthood resulting in metabolic disorders. Indeed, it has been argued that hyperactive and impulsive symptoms may lead to abnormal eating behaviors and binge eating, which in turn may lead to obesity and other metabolic disorders [42]. Also, it is important to note that metabolic disorders are commonly regarded as risk factors for CVDs disorders [5, 6], and they may stand in a causal pathway between ADHD symptoms and CVDs. Thus, eating behaviors, in addition to other lifestyle factors and psychiatric comorbidity, should be addressed within preventative strategies for cardiometabolic conditions in adults with ADHD.

Overall, we showed that the associations of ADHD with cardiometabolic disorders were mostly independent of the ADHD subscale used (i.e., inattention and hyperactivity/impulsivity subscales), definition of ADHD, and sex, and they remained consistent when we limited the analysis to same-sex dizygotic twin pairs.

### Study limitations

The results of the current study need to be considered in light of several potential study limitations. Namely, the utilized ADHD scale covered only currently present symptoms, without addressing whether ADHD symptoms were present since childhood and whether they were accompanied by functional impairments, which are also required as diagnostic criteria by the DSM-IV. This is important, as in adults, ADHD symptoms may resemble the symptoms of other psychiatric disorders, such as anxiety or depression [43], or neurodegenerative disorders [44], which may lead to misdiagnosis in both directions. Nevertheless, our sensitivity analyses which investigated the risks of CVDs and metabolic disorders considering only those who were clinically diagnosed with ADHD confirmed the same pattern of the associations identified in the main analysis.

Furthermore, it may be difficult to discern the effects of ADHD symptoms and ADHD medications in relation to the risk of CVDs. ADHD stimulant medications have been shown to affect blood pressure and heart rate and they are prescribed with clinical precautions regarding the cardiovascular risks [45]. However, only 0.75% of our study population received ADHD medication, and a recent meta-analysis has indicated that there is no statistically significant association between ADHD medication use and CVDs [46]. It has also been shown that the associations between ADHD genetic liability and cardiometabolic risks remain even in the absence of medical treatment for ADHD [13].

Furthermore, all variables assessed using the STAGE interview/questionnaire were based on self-assessment. There are clear advantages to this approach. It allows assessment of large study populations at lower costs than assessment by trained professionals and provides wider coverage of affected individuals compared to using data from electronic health registers, which only capture individuals who receive a formal diagnosis and in Sweden only cover specialist healthcare visits (i.e., more severe patients). Furthermore, there is very poor coverage of lifestyle factors in electronic health registers, such as obesity, smoking, and physical activity. Finally, using continuous scales of ADHD symptoms rather than clinical diagnoses allows for more detailed data and in turn provides greater statistical power, which is needed in within-twin analyses. On the other hand, measurements of health-related variables based on self-assessment may arguably be unreliable [47]. For instance, it has been shown that self-reports in adults may underestimate ADHD symptoms compared to parent reports [48]. This issue was partially addressed by conducting an additional analysis of the association of clinically diagnosed ADHD with cardiometabolic disorders. Furthermore, it is reassuring that our findings on the link between ADHD and cardiometabolic disorders are largely in line with past findings using electronic health records [8–10, 13, 14]. Future studies using health-related data assessed by trained professionals are, however, still needed to replicate our findings.

Furthermore, about 5% of the study population had self-reported BMI categorized as obese at the time of STAGE, although only 1.9% received a clinical diagnosis of obesity during the follow-up. Thus, individuals diagnosed with obesity were likely already obese at the start of the follow-up and probably only those with associated health problems sought health care and received a diagnosis of obesity. Consequently, the outcome of obesity in the current study probably covers individuals with the most severe clinical presentations of the condition.

In the current paper, the included covariates may likely have mediated the association between ADHD symptoms and cardiometabolic disorders. However, the covariates were assessed at the same time as the exposure (i.e., ADHD symptoms), and we only adjusted our analyses for these covariates to observe whether the associations changed after their inclusion in the model. To more thoroughly investigate whether they have the role of mediators and to provide estimates of the presumed mediators’ effects on the associations, future studies need to consider assessing these variables after the assessment of ADHD symptoms, as well as conducting a formal mediation analysis, such as the causal mediation analysis [49].

Additionally, the oldest individuals in our cohort were only 59 years old at the end of follow-up (i.e., end of 2018). The incidence of cardiometabolic disorders peaks after the age of 60–65 [50, 51], and we may need a longer follow-up of these individuals to a more advanced age to fully understand the associations with ADHD symptoms and the underlying mechanisms. Furthermore, as the non-responders in the STAGE were more likely to be male, have at least one parent born outside Sweden, and be diagnosed with a psychiatric disorder, the generalizability of our study findings may be limited, as non-responders might have had higher levels of ADHD symptoms than responders [26]. Future studies should investigate the associations of ADHD symptoms with cardiometabolic disorders in study samples with a higher representation of these demographic groups.

Finally, due to the discrepancies in the analytical sample sizes for the analysis at the whole study population level, after adjustments for covariates, and within dizygotic/monozygotic twins and considering the overall small effect sizes of the associations with overlapping confidence intervals across analyses, we cannot rule out that the attenuation of the associations after the adjustments for covariates and for familial factors shared between monozygotic twins may be partially due to the loss of statistical power. Nevertheless, when it comes to the associations between ADHD symptoms and CVDs, we found a more pronounced and statistically significant attenuation in the strength of the association within monozygotic twins compared to the analysis within dizygotic twins. This was not the case for the associations with metabolic disorders, although the decrease in the number of individuals with disease outcomes was equivalent for the analyses with both CVDs and metabolic disorders. Thus, our results may indicate genetic confounding of the association between ADHD symptoms and CVDs.

## Conclusions

Using a longitudinal population-based study design, we found that ADHD symptom score is associated with a subsequent increased risk of both CVDs and metabolic disorders. Our findings also suggest that this risk may be explained by adverse life-course factors associated with ADHD—inadequate educational attainment, unhealthy lifestyle factors, and comorbid psychiatric disorders. Thus, it may be beneficial for future prevention programs to target these potentially modifiable risk factors in adults with ADHD. Additionally, our findings indicated that the association between ADHD symptoms and the risk of CVDs might be confounded by genetic factors to some extent. Yet, we did not find as strong evidence for shared genetic factors explaining the association with metabolic disorders. Future studies are needed to investigate the identified associations at the level of individual cardiometabolic disorder categories, with longer follow-up of potential mediators and cardiometabolic outcomes to a more advanced older age.

### Supplementary Information

**Additional file 1:** **Table S1.** Diagnostic codes for the cardiovascular and metabolic disorders included in the study, according to the International Classification of Diseases 10th revisions (ICD-10) and medication prescriptions coded according to the Anatomical Therapeutic Classification (ATC). **Table S2.** Incidence of clinical diagnoses/dispensed medication prescriptions for cardiovascular and metabolic disorders as distinct categories, and associations with ADHD score, expressed as hazard ratios (HR) with 95% confidence intervals (CI), adjusted for birth year and sex. **Table S3.** Associations of scores on the inattention (IA) and hyperactivity/impulsivity (HI) subscales, with cardiovascular (CVD) and metabolic disorders, presented as hazard ratios (HR) with 95% confidence intervals (CI). **Table S4.** Frequencies for clinically diagnosed individuals and individuals with research diagnosis of ADHD. **Table S5.** Associations of ADHD score with cardiovascular disorders (CVDs) and metabolic disorders, as hazard ratios with 95% confidence intervals for men. **Table S6.** Associations of ADHD score with cardiovascular disorders (CVDs) and metabolic disorders, as hazard ratios with 95% confidence intervals for women. **Table S7.** Associations of ADHD score with cardiovascular disorders (CVDs) and metabolic disorders, as hazard ratios with 95% confidence intervals (CI) for monozygotic (MZ) twin pairs, and dizygotic (DZ) twin pairs with the same-sex female/male twin pairs. Supplementary methods. Description of DSM-IV criteria for psychiatric comorbidities. **Fig. S1-S6.** Natural cubic spline function to model non-linear associations between ADHD symptoms and cardiometabolic disorders. **Table S8.** Associations between quartiles, the 90th and 99th percentile of ADHD scores and the risk of cardiometabolic outcomes.

## Data Availability

Data may be obtained from a third party and are not publicly available. The Public Access to Information and Secrecy Act in Sweden prohibits individual-level data to be publicly available. Researchers who are interested in replicating this study can apply for individual level data at The Swedish Twin Registry, managed by Karolinska Institutet: https://strdata.se/

## Abbreviations

ADHD: Attention-deficit/hyperactivity disorder
CVD: Cardiovascular disorder
STAGE: Study of Twin Adults: Genes and Environment
HR: Hazard ratio
CI: Confidence interval
NPR: National Patient Register
ICD: International Statistical Classification of Diseases and Related Health Problems
PDR: Prescribed Drug Register
ATC: Anatomical Therapeutic Classification
BMI: Body mass index
DSM: Diagnostic and Statistical Manual of Mental Disorders
SD: Standard deviation
