# Supplementary material for: Attention-deficit/hyperactivity disorder symptoms and subsequent cardiometabolic disorders in adults: investigating underlying mechanisms using a longitudinal twin study
Source: BMC Med. 2023 Nov 22;21:452. doi: 10.1186/s12916-023-03174-1 (PMC10664476; doi:10.1186/s12916-023-03174-1)
Supplement: Supplementary file 1 — Additional file 1: Table S1. Diagnostic codes for the cardiovascular and metabolic disorders included in the study, according to the International Classification of Diseases 10th revisions (ICD-10) and medication prescriptions coded according to the Anatomical Therapeutic Classification (ATC). Table S2. Incidence of clinical diagnoses/dispensed medication prescriptions for cardiovascular and metabolic disorders as distinct categories, and associations with ADHD score, expressed as hazard ratios (HR) with 95% confidence intervals (CI), adjusted for birth year and sex. Table S3. Associations of scores on the inattention (IA) and hyperactivity/impulsivity (HI) subscales, with cardiovascular (CVD) and metabolic disorders, presented as hazard ratios (HR) with 95% confidence intervals (CI). Table S4. Frequencies for clinically diagnosed individuals and individuals with research diagnosis of ADHD. Table S5. Associations of ADHD score with cardiovascular disorders (CVDs) and metabolic disorders, as hazard ratios with 95% confidence intervals for men. Table S6. Associations of ADHD score with cardiovascular disorders (CVDs) and metabolic disorders, as hazard ratios with 95% confidence intervals for women. Table S7. Associations of ADHD score with cardiovascular disorders (CVDs) and metabolic disorders, as hazard ratios with 95% confidence intervals (CI) for monozygotic (MZ) twin pairs, and dizygotic (DZ) twin pairs with the same-sex female/male twin pairs. Supplementary methods. Description of DSM-IV criteria for psychiatric comorbidities. Fig. S1-S6. Natural cubic spline function to model non-linear associations between ADHD symptoms and cardiometabolic disorders. Table S8. Associations between quartiles, the 90th and 99th percentile of ADHD scores and the risk of cardiometabolic outcomes. [file 12916_2023_3174_MOESM1_ESM.docx]

**Supplementary information**

**Attention-deficit/hyperactivity disorder symptoms and subsequent cardiometabolic disorders in adults: investigating underlying mechanisms using a longitudinal twin study**

Maja Dobrosavljevic, Ralf Kuja-Halkola, Lin Li, Zheng Chang, Henrik Larsson, Ebba Du Rietz

**Content**

**Table S1.** Diagnostic codes for the cardiovascular and metabolic disorders included in the study, according to the International Classification of Diseases 10^th^ revisions (ICD-10) and medication prescriptions coded according to the Anatomical Therapeutic Classification (ATC) ***Page 2***

**Table S2.** Incidence of clinical diagnoses/dispensed medication prescriptions for cardiovascular and metabolic disorders as distinct categories, and associations with ADHD score, expressed as hazard ratios (HR) with 95% confidence intervals (CI), adjusted for birth year and sex ***Page 3***

**Table S3.** Associations of scores on the inattention (IA) and hyperactivity/impulsivity (HI) subscales, with cardiovascular (CVD) and metabolic disorders, presented as hazard ratios (HR) with 95% confidence intervals (CI) ***Page 4***

**Table S4.** Frequencies for clinically diagnosed individuals and individuals with research diagnosis of ADHD ***Page 5***

**Table S5.** Associations of ADHD score with cardiovascular disorders (CVDs) and metabolic disorders, as hazard ratios with 95% confidence intervals for men ***Page 5***

**Table S6.** Associations of ADHD score with cardiovascular disorders (CVDs) and metabolic disorders, as hazard ratios with 95% confidence intervals for women ***Page 6***

**Table S7.** Associations of ADHD score with cardiovascular disorders (CVDs) and metabolic disorders, as hazard ratios with 95% confidence intervals (CI) for monozygotic (MZ) twin pairs, and dizygotic (DZ) twin pairs with the same-sex female/male twin pairs ***Page 6***

**Supplementary methods.** Description of DSM-IV criteria for psychiatric comorbidities ***Page 7***

**Fig. S1-S6.** Natural cubic spline function to model non-linear associations between ADHD symptoms and cardiometabolic disorders ***Page 8***

**Table S8.** Associations between quartiles, the 90th and 99th percentile of ADHD scores and the risk of cardiometabolic outcomes ***Page 10***

**Table S1.** Diagnostic codes for the cardiovascular and metabolic disorders included in the study, according to the International Classification of Diseases 10^th^ revisions (ICD-10) and medication prescriptions coded according to the Anatomical Therapeutic Classification (ATC).

|  | **ICD-10** | **ATC** |
| --- | --- | --- |
| **Ischemic heart disease** | **I20-I25** | **C01** |
| Acute coronary syndrome (ACS) | I21-I24, I25.2, I20.0 |  |
| Chronic coronary syndrome (without ACS) | I20.1-I20.9, I25.1, I25.5-I25.9 |  |
| **Cerebrovascular disease** | **I60-I69** | - |
| Subarachnoidal bleeding | I60 |  |
| Hemorrhagic stroke | I61-I62 |  |
| Ischemic stroke | I63-I64 |  |
| Other cerebrovascular disease | I65-I69 |  |
| **Venous thrombo-embolism** | **I26, I80** | - |
| Deep vein thrombosis | I80 |  |
| Pulmonary emboli | I26 |  |
| **Heart failure** | **I50** | **C01A** |
| **Takyarrhythmias:** | **I46, I47.0, I47.1, I47.2, I48, I49.0, I49.8** | **C01A, C01B** |
| Atrial fibrillation/fludder | I48 |  |
| Supraventricular tachycardia | I47.1 |  |
| Ventricular tachycardia | I47.0, I47.2, I49.0, I49.8 |  |
| Cardiac arrest | I46 |  |
| **Hypertensive diseases** | **I10-I15** | **C02, C03, C07, C08, C09** |
| **Type 2 Diabetes** | **E11** | **A10A, A10B** |
| **Obesity** | **E65-E66** | **-** |
| **Hyperlipidaemia** | **E78** | **C10** |

**Table S2.** Incidence of clinical diagnoses/dispensed medication prescriptions for cardiovascular and metabolic disorders as distinct categories, and associations with ADHD score, expressed as hazard ratios (HR) with 95% confidence intervals (CI), adjusted for birth year and sex, within the total study population (N=10,394)

|  | **N (%)** | **ADHD score**  **HR 95% CI** | **P value** |
| --- | --- | --- | --- |
| **Ischemic heart disease** | 64 (0.62) | 1.05 (1.00, 1.10) | 0.049 |
| **Cerebrovascular disease** | 51 (0.49) | 1.01 (0.95, 1.08) | 0.778 |
| **Venous thrombo-embolism** | 99 (0.95) | 1.01 (0.97, 1.05) | 0.717 |
| **Heart failure** | 24 (0.23) | 0.99 (0.90, 1.09) | 0.840 |
| **Tachyarrhythmias** | 94 (0.90) | 1.00 (0.96, 1.05) | 0.916 |
| **Hypertensive diseases** | 293 (2.82) | 1.02 (1.00, 1.05) | 0.082 |
| **Dispensed medication prescription for CVD** | 1041 (10.02) | 1.03 (1.01, 1.04) | <0.001 |
| **Any CVD** | 1313 (12.63) | 1.02 (1.01, 1.04) | <0.001 |
| **Type 2 diabetes** | 59 (0.57) | 1.03 (0.98, 1.08) | 0.256 |
| **Obesity** | 199 (1.91) | 1.05 (1.02, 1.07) | <0.001 |
| **Hyperlipidaemia** | 86 (0.83) | 1.05 (1.01, 1.09) | 0.022 |
| **Dispensed medication prescription for metabolic disorders** | 566 (5.45) | 1.03 (1.01, 1.05) | <0.001 |
| **Any metabolic disorder** | 728 (7.00) | 1.03 (1.02, 1.05) | <0.001 |

**Table S3.** Associations of scores on the inattention (IA) and hyperactivity/impulsivity (HI) subscales, with cardiovascular (CVD) and metabolic disorders, presented as hazard ratios (HR) with 95% confidence intervals (CI)

|  | **CVD**  **HR (95% CI)** | | **Metabolic disorders**  **HR (95% CI)** | |
| --- | --- | --- | --- | --- |
| **IA**  Adjustment for birth year and sex^1^  Additional adjustment for covariates^2^ | **1.04 (1.02, 1.06), p<0.001**  1.01 (0.98, 1.03), p=0.594 | | **1.06 (1.03, 1.08), p<0.001**  1.02 (0.99, 1.05), p=0.273 | |
| Additional adjustment for familial factors shared between twins in: | | | | |
| Dizygotic twin pairs^3^ | **1.07 (1.03, 1.12),**  **p=0.001** | *χ*^2^=3.62, p=0.057 | 1.05 (1.00, 1.11)  p=0.061 | *χ*^2^=0.39, p=0.533 |
| Monozygotic twin pairs^4^ | 1.00 (0.94, 1.06), p=0.975 |  | 1.02 (0.95, 1.10), p=0.508 |  |
| **HI**  Adjustment for birth year and sex^1^  Additional adjustment for covariates^2^ | **1.03 (1.01, 1.05), p=0.007**  1.00 (0.98, 1.03), p=0.698 | | **1.04 (1.02, 1.07), p<0.001**  1.01 (1.00, 1.05), p=0.062 | |
| Additional adjustment for familial factors shared between twins in: | | | | |
| Dizygotic twin pairs^3^ | **1.05 (1.01, 1.10),**  **p=0.014** | *χ*^2^=2.33, p=0. 126 | **1.06 (1.01, 1.12),**  **p=0.031** | *χ*^2^=0.34, p=0.558 |
| Monozygotic twin pairs^4^ | 1.00 (0.94, 1.06),  p=0.907 |  | **1.09 (1.01, 1.18), p=0.027** |  |

^1^ N=10,394 total included in the analysis, with CVD N=1313, with metabolic disorders N=728

^2^Covariates: educational attainment, lifestyle, and psychiatric disorders, N= 8305 total included in the analysis for the association with CVD, N with CVD 1007; N=8434 total included in the analysis for the association with metabolic disorders, N with 587, analysis for the association with metabolic disorders was not adjusted for BMI.

^3^N= 5,618 total included in the analysis, with CVD N=747, with metabolic disorder N=435

^4^N=4,766 total included in the analysis, with CVD N=566, with metabolic disorders N=293

**Table S4.** Frequencies for clinically diagnosed individuals and individuals with research diagnosis of ADHD.

| **Definition of ADHD** | **Whole sample**  **N=** **10,394** | **Monozygotic twins**  **N=** **4,776** | **Dizygotic twins**  **N=5,618** |
| --- | --- | --- | --- |
|  | **N (%)** | | |
| **Clinical diagnosis** | **79 (0.76)** | **34 (0.71)** | **45 (0.80)** |
| Research diagnosis: primarily inattentive subtype | 171 (1.65) | 78 (1.63) | 93 (1.66) |
| Research diagnosis: primarily hyperactive/impulsive subtype | 104 (1.00) | 37 (0.77) | 67 (1.19) |
| Research diagnosis: combined subtype | 36 (0.35) | 19 (0.40) | 17 (0.30) |
| **Research diagnosis: any** | **311 (2.99)** | **134 (2.81)** | **177 (3.15)** |

**Table S5.** Associations of ADHD score with cardiovascular disorders (CVDs) and metabolic disorders, as hazard ratios with 95% confidence intervals for men

|  | **CVD**  **HR (95% CI)** | | **Metabolic disorders**  **HR (95% CI)** | |
| --- | --- | --- | --- | --- |
| Adjustment for birth year and  sex^1^ | **1.02 (1.00, 1.04), p=0.014** | | **1.03 (1.01, 1.05), p=0.003** | |
| Additional adjustment for covariates^2^ | 1.01 (0.98, 1.03), p=0.646 | | 1.01 (0.98, 1.03), p=0.599 | |
| Additional adjustment for familial factors shared between twins in: | | | | |
| Dizygotic twin pairs^3^  Monozygotic twin pairs^4^ | **1.12 (1.04, 1.22), p=0.003**  0.99 (0.93, 1.07),  p=0.884 | χ2=5.12, p=0.024 | 1.02 (0.95, 1.11),  p=0.544  1.06 (0.98, 1.15),  p=0.154 | χ2=0.37,  p= 0.545 |

^1^ N=3985 total included in the analysis with individuals from both complete and incomplete twin pairs, with CVD N=466, with metabolic disorders N=312

^2^Covariates: educational attainment, lifestyle, and psychiatric disorders, N= 3131, total included in the analysis for the association with CVD, N with CVD is 358; N=3150 total included in the analysis for the association with metabolic disorders, N with metabolic disorders is 250; analysis for the association with metabolic disorders was not adjusted for BMI.

^3^N= 1036 total from complete twin pairs, with CVD N=120, with metabolic disorder N=83

^4^N= 1620 total from complete twin pairs, with CVD N=165, with metabolic disorders N=95

**Table S6.** Associations of ADHD score with cardiovascular disorders (CVDs) and metabolic disorders, as hazard ratios with 95% confidence intervals for women

|  | **CVD**  **HR (95% CI)** | | **Metabolic disorders**  **HR (95% CI)** | |
| --- | --- | --- | --- | --- |
| Adjustment for birth year and  sex^1^ | **1.02 (1.01, 1.04), p=0.004** | | **1.04 (1.02, 1.05), p<0.001** | |
| Additional adjustment for covariates^2^ | 1.00 (0.98, 1.02), p=0.573 | | **1.02 (1.00, 1.04), p=0.044** | |
| Additional adjustment for familial factors shared between twins in: | | | | |
| Dizygotic twin pairs^3^  Monozygotic twin pairs^4^ | **1.05 (1,00, 1.10), p=0.034**  1.00 (0.96, 1.04),  p=0.973 | χ2=2.43, p=0.118 | 1.05 (1.00, 1.11),  p=0.062  1.04 (0.98, 1.10),  p=0.227 | χ2=0.18,  p= 0.667 |

^1^ N=6409 total included in the analysis with individuals from both complete and incomplete twin pairs with CVD N=847, with metabolic disorders N=416

^2^Covariates: educational attainment, lifestyle, and psychiatric disorders, N= 5174, total included in the analysis for the association with CVD, N with CVD is 642; N=5284 total included in the analysis for the association with metabolic disorders, N with metabolic disorders is 337; analysis for the association with metabolic disorders was not adjusted for BMI.

^3^N=1926 total from complete twin pairs, with CVD N=259, with metabolic disorder N= 129

^4^N= 3156 total from complete twin pairs, with CVD N=401, with metabolic disorders N=198

**Table S7.** Associations of ADHD score with cardiovascular disorders (CVDs) and metabolic disorders, as hazard ratios with 95% confidence intervals (CI) adjusted for familial factors shared between twins in monozygotic and same-sex dizygotic twin pairs.

|  | CVD  HR (95% CI) | *χ*^2^/p value | Metabolic disorders  HR (95% CI) | *χ*^2^/p value |
| --- | --- | --- | --- | --- |
| Dizygotic twins^1^  Monozygotic twins^2^ | **1.07 (1.03, 1.11),**  **p<0.001**  1.00 (0.97, 1.03), p=0.968 | **6.56/0.010** | 1.04 (1.00, 1.09),  p=0.057  1.04 (1.00, 1.09), p=0.069 | 0.00/0.997 |

^1^N= 2,962 total included in the analysis, with CVD N=379, with metabolic disorders N=212

^2^N=4,766 total included in the analysis, with CVD N=566, with metabolic disorders N=293

**Supplementary methods.** Description of DSM-IV criteria for psychiatric comorbidities

To receive a research diagnosis of major depression, (1) depressed mood, or (2) loss of interest or pleasure needed to be present, as well as at least four symptoms out of the following symptoms: (3) tiredness, fatigue, or low energy, (4) significant unintentional weight loss/gain or decrease/increase in appetite, (5) sleep disturbance (insomnia or hypersomnia), (6) impaired ability to think, concentrate, or make decisions, (7) psychomotor changes (agitation or retardation), (8) feeling of worthlessness or guilt, and (9) recurrent thoughts of death. If both (1) and (2) were present, at least three out of the remaining symptoms needed to be present as well. Additionally, these symptoms must cause significant distress or impairment, and should not follow a loss of a loved one.

Generalized anxiety disorder was identified as excessive anxiety and worry, accompanied by at least three out of the following symptoms: (1) restlessness, (2) irritability, (3) fatigue, (4) sleep disturbance, (5) muscle tension, and (6) difficulty concentrating.

Alcohol dependence was identified when at least three out of the following seven symptoms were reported: (1) tolerance, as needing increased amounts to achieve the same effect, or diminished effect with the same amount of alcohol, (2) symptoms of withdrawal or avoidance of withdrawal by continued use of the substance, (3) substance consumption in larger amounts or for a longer time than it was intended to, (4) a persistent desire or unsuccessful efforts to control substance use, (5) a great amount of time is spent to obtain or use the substance or recover from its effects, (6) relevant social, occupation or recreational activities are reduced or given up due to substance use, (7) continued use of alcohol despite knowledge of having adverse effects due to the substance use.

**Fig. S1-S6: Natural cubic spline function to model non-linear associations between ADHD symptoms and cardiometabolic disorders (black line, with 95% CI in gray), presented together with the linear model (in green)**

**
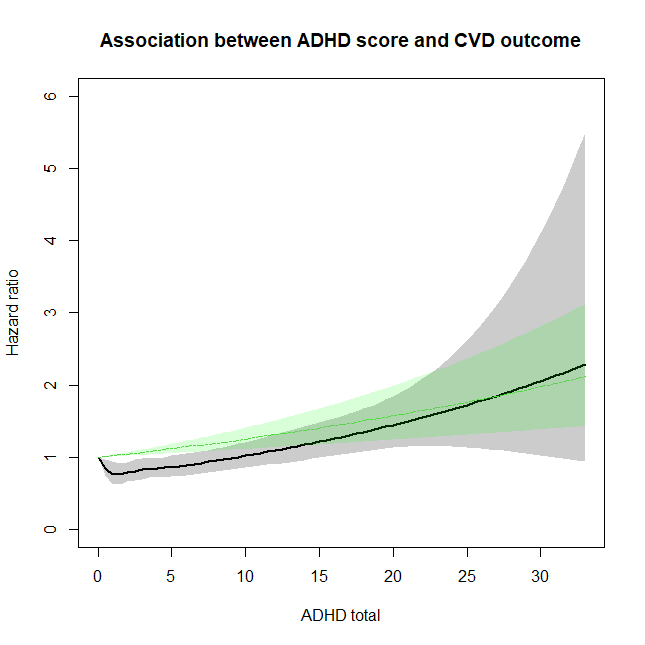

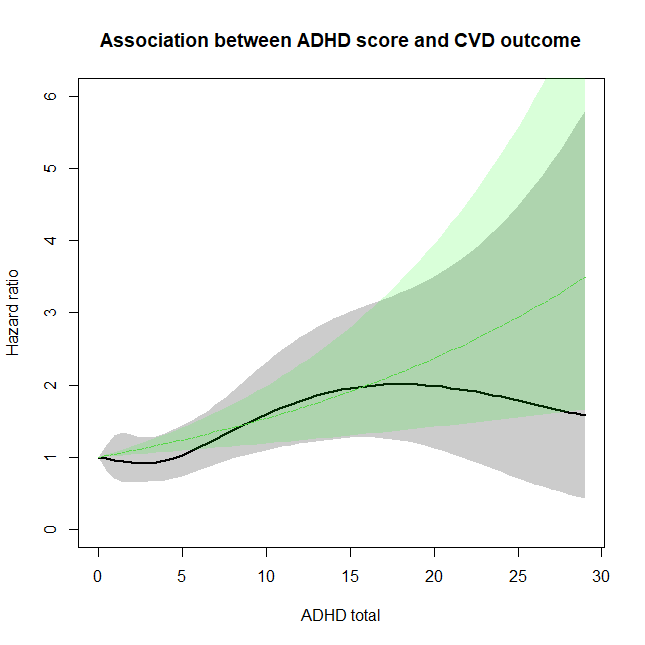

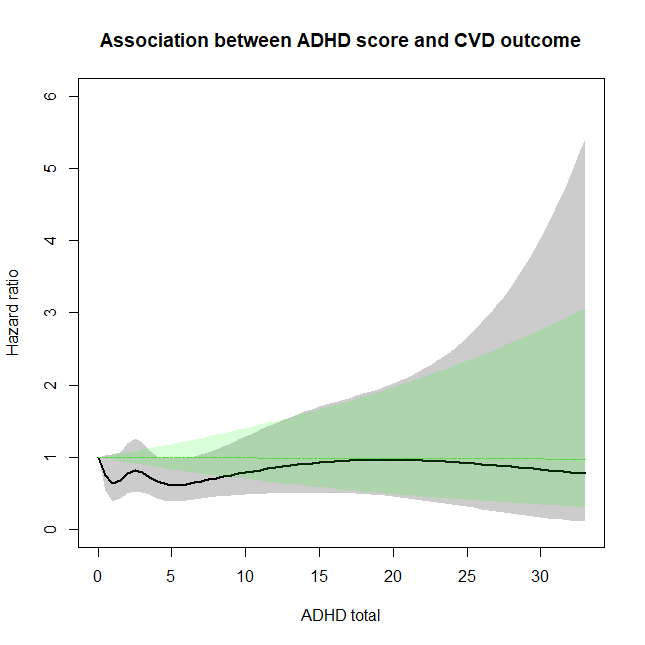
**

**Fig. S3.** The analysis adjusted for familial factors shared by monozygotic twins (df=5)

C index for linear model = 0.491

C index for 4-knot spline regression=0.586

**Fig. S2.** The analysis adjusted for familial factors shared by dizygotic twins (df=4)

C index for linear model = 0.56

C index for 3-knot spline regression=0.544

**Fig. S1.** The analysis at the level of the whole study population (degrees of freedom (df)=5)

Concordance (C) index for linear model=0.583

C index for 4-knot spline regression=0.588

Note: ADHD total is the score on the total ADHD scale

**
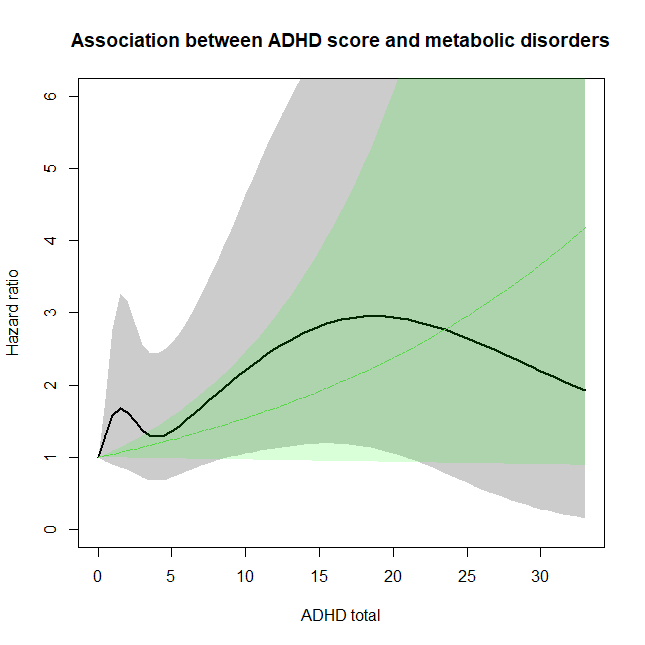

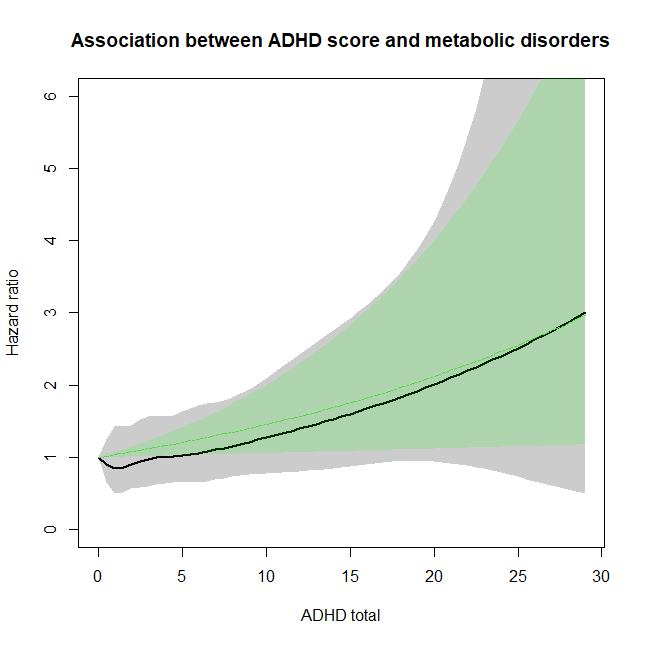

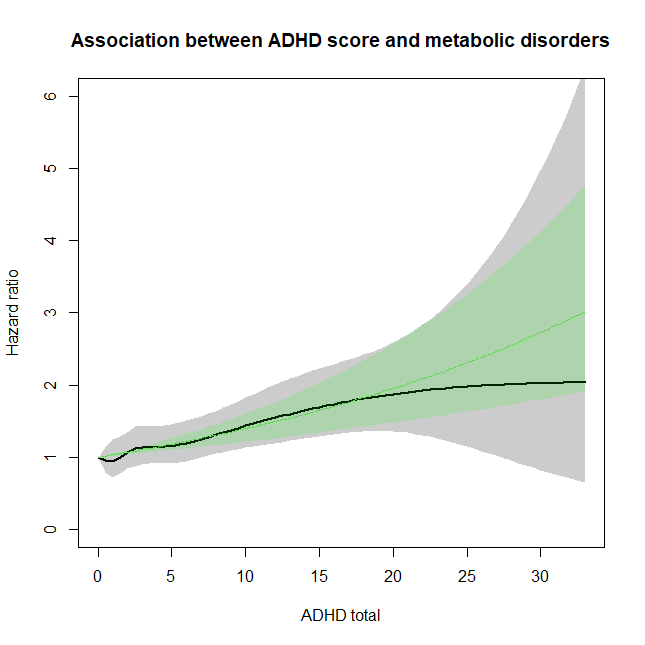
**

**Fig. S4.** The analysis at the level of the whole study population (df=5)

C index for linear model=0.583

C index for 4-knot spline regression=0.6

**Fig. S6.** The analysis adjusted for familial factors shared by monozygotic twins (df=4)

C index for linear model=0.565

C index for 3-knot spline regression=0.556

**Fig. S5.** The analysis adjusted for familial factors shared by dizygotic twins (df=5)

C index for linear model=0.551

C index for 4-knot spline regression=0.562

**Table S8.** Associations between quartiles (25^th^, 50^th^ and 75^th^ percentile) and the 90^th^ and 99^th^ percentile of ADHD scores and the risk of cardiometabolic outcomes at the level of the whole study population (N=10,394)

| Percentile | ADHD score | CVD HR (95% CI) | Metabolic disorders |
| --- | --- | --- | --- |
| 25 | 1 | 0.77 (0.63, 0.94) | 0.94 (0.72, 1.25) |
| 50 | 3 | 0.83 (0.70, 0.98) | 1.14 (0.90, 1.44) |
| 75 | 7 | 0.92 (0.78, 1.09) | 1.25 (0.99, 1.57) |
| 90 | 11 | 1.06 (0.89, 1.26) | 1.49 (1.16, 1.92) |
| 99 | 21 | 1.50 (1.15, 1.95) | 1.90 (1.34, 2.70) |
